# Use of the particle agglutination/particle agglutination inhibition test for antigenic analysis of SARS‐CoV‐2

**DOI:** 10.1111/irv.13093

**Published:** 2023-02-06

**Authors:** Jun Kobayashi, Shutoku Matsuyama, Masayuki Shirakura, Tomoko Arita, Yasushi Suzuki, Hideki Asanuma, Shinji Watanabe, Hideki Hasegawa, Kazuya Nakamura

**Affiliations:** ^1^ Laboratory of Acute Respiratory Virus, Research Center for Influenza and Respiratory Viruses National Institute of Infectious Diseases Tokyo Japan; ^2^ Laboratory of Influenza Virus Surveillance, Research Center for Influenza and Respiratory Viruses National Institute of Infectious Diseases Tokyo Japan; ^3^ Laboratory of Vaccine Seed Virus Development, Research Center for Influenza and Respiratory Viruses National Institute of Infectious Diseases Tokyo Japan

**Keywords:** antigenic analysis, particle agglutination, particle agglutination inhibition, SARS‐CoV‐2

## Abstract

**Background:**

The antigenicity of SARS‐CoV‐2 is a critical issue for the effectiveness of the vaccine, and thus, it should be phenotypically evaluated by serological assays as new field isolates emerge. The hemagglutination/hemagglutination inhibition (HA/HI) tests are well known as a representative method for antigenic analysis of influenza viruses, but SARS‐CoV‐2 does not agglutinate human or guinea pig red blood cells. Therefore, the antigenic analysis requires complicated cell‐based assays using special equipment such as plate reader or ELISPOT analyzer.

**Methods:**

Based on the HA/HI tests for influenza viruses, we developed the particle agglutination/particle agglutination inhibition (PA/PAI) test to easily and rapidly quantify the virus and antibody using human angiotensin‐converting enzyme 2 (hACE2)‐bound latex beads. The virus titers were determined by mixing the beads and the virus from culture supernatant, settling it overnight, and then observing the sedimentation/agglutination pattern (PA test). The neutralization antibody titers were determined by mixing virus‐infected hamster antisera in addition to the beads and virus (PAI test).

**Results:**

The PA titer was positively correlated with the plaque‐forming units. The PAI titer using the hamster antisera clearly revealed the antigenic difference between the omicron and previous variants. The antigenic differences were supported by the results shown in other methods.

**Conclusions:**

The PAI test is an easy and rapid method to analyze the antigenicity of SARS‐CoV‐2.

## 1 INTRODUCTION

SARS‐CoV‐2, which causes COVID‐19, has infected over 500 million people and killed over 6 million, despite the production of over 11 billion doses of vaccine as of April 2022 (WHO COVID‐19 Dashboard, https://covid19.who.int/). The pandemic of SARS‐CoV‐2, with its continuously evolving viral properties, has become a major public health concern that adds to the existing concern regarding influenza virus pandemics/epidemics. In particular, the omicron variants that emerged in November 2021 have a higher number of mutations than the previous variants and are considered to have relatively mild symptoms, but to be highly infectious. Furthermore, the omicron variants are resistant to immunity raised by previous variants^1^, ^2^ and thus have caused the largest number of infections.

To predict the antigenicity and other viral properties of each isolate, a large number of the viral genomes have been sequenced using next‐generation sequencers and registered (GISAID; https://www.gisaid.org/).^3^ However, the antigenicity of each variant must also be phenotypically evaluated by serological studies. The neutralization assay^4^ is one of the methods to analyze the antigenicity of SARS‐CoV‐2 isolates, but it is a time‐consuming and complicated cell‐based assay. A simpler enzyme‐linked immunosorbent assay (ELISA) method without cells has also been reported,^5^ but it requires artificially modified/purified proteins, such as the human angiotensin‐converting enzyme 2 (hACE2) and the receptor binding domain of the SARS‐CoV‐2 spike protein, and a plate reader.

The antigenicity of influenza virus has been easily and rapidly analyzed by means of hemagglutination (HA)/hemagglutination inhibition (HI) tests.^6^ SARS‐CoV‐2 shows the hemadsorption activity with human erythrocytes on the virus‐infected Vero cells, but does not exhibit direct HA activity using human and guinea pig erythrocytes.^7^ In addition, there are increasing limitations on the use of blood cells due to various issues, such as animal ethics.^8^ Therefore, the HA/HI tests are not applicable for SARS‐CoV‐2, and a surrogate for blood cells is required.

In this study, we established particle agglutination (PA)/particle agglutination inhibition (PAI) methods using hACE2‐bound latex beads as a surrogate for blood cells, utilizing the phenomenon that SARS‐CoV‐2 interacts with hACE2 via its spike protein.^9^ These methods enable easy and rapid measurement of SARS‐CoV‐2 titer and antibody titer against the virus as well as antigenic analysis without special equipment.

## 2 METHODS

### 2.1 SARS‐CoV‐2 culture

Eighty to ninety‐five percent confluent Vero E6 cells expressing transmembrane protease serine 2 (TMPRSS2)^10^ were infected with SARS‐CoV‐2 strains using DMEM supplemented with 5% fetal bovine serum and penicillin/streptomycin and then cultured at 37°C under 5% CO_2_. The following isolates, which were mainly prevalent in Japan, were used (information on mutations is summarized in Table S1). WK‐521 (Pango lineage: A), DP15‐037 (Pango lineage: A), QH‐329‐037 (Pango lineage: B.1), QHN001 (alpha variant, Pango lineage: B.1.1.7), TY8‐612 (beta variant, Pango lineage: B.1.351), TY11‐927 (delta variant, Pango lineage: B.1.617.2), TY29‐009 (delta variant, Pango lineage: B.1.617.2), and TY11‐330 (kappa variant, Pango lineage: B.1.617.1) isolates were harvested 24 h after infection by centrifugation at 1000 rpm for 5 min at room temperature. TY38‐873 (omicron variant, Pango lineage: BA.1), TY38‐871 (omicron variant, Pango lineage: BA.1), and TY40‐385 (omicron variant, Pango lineage: BA.2) isolates were harvested 48 h after infection by centrifugation. The harvested viruses were stored at −80°C until use.

### 2.2 Beads (particle) preparation

Deep blue‐dyed latex beads, 0.8 μm in average diameter, were purchased from Sigma‐Aldrich (catalog no. L1398). 0.5 ml of 2.5% (w/v) beads was centrifuged at 2400 g for 10 min at room temperature, and the beads were recovered as precipitates. The beads were washed twice with 1 ml PBS (Takara; catalog no. T900) using centrifugation. The washed beads were centrifuged and resuspended in 0.25 ml of 25 mM MES‐NaOH, pH 6.0. The resuspended beads were centrifuged and mixed with 2.5 ml of 25 mM MES‐NaOH, pH 6.0 containing 100 μg hACE2 (GeneTex; catalog no. GTX01550‐pro) for 24 h at 4°C using a rotator. The hACE2‐bound beads were centrifuged at 2400 g for 10 min at 4°C and washed twice with 1 ml PBS. The OD_280_ of the hACE2 solution was measured before and after mixing of hACE2 solution and the beads. The beads were blocked with 0.75 ml of PBS with 3% bovine serum albumin (BSA, Sigma‐Aldrich; catalog no. A9418) for 30 min at room temperature. The blocked beads were stored in 0.5 ml of PBS with 1% BSA (final 2.5% (w/v) beads concentration) at 4°C until use.

### 2.3 PA test

2.5% (w/v) hACE2‐beads were diluted to 0.003%–0.8% (w/v) with PBS supplemented with 1% BSA. Based on sedimentation/agglutination patterns and detection sensitivity of the virus, the optimum concentration was set at 0.01%. 50 μl aliquots of a twofold dilution series of SARS‐CoV‐2 variants were prepared in a 96‐well plate with PBS. Fifty microliters of 0.01% (w/v) hACE2‐beads was added to each well. After overnight settling at room temperature, sedimentation/agglutination patterns were observed. A mixture of 50 μl of PBS and 50 μl of the beads was used as the sedimentation (no‐agglutination) control.

### 2.4 Titration of SARS‐CoV‐2

The titers of SARS‐CoV‐2 used in this study were determined either by plaque assay or by 50% tissue culture infectious dose (TCID_50_). For the plaque assay, monolayers of VeroE6/TMPRSS2 cells grown in a 96‐well plate were infected with serially diluted culture supernatants of SARS‐CoV‐2, cultured in high‐glucose Dulbecco's modified Eagle's medium (DMEM; Sigma‐Aldrich) containing 2.5% carboxymethyl cellulose at 37°C under 5% CO_2_ for 3 days and then fixed with 4% paraformaldehyde and stained with crystal violet. Emergent plaques were counted using an optical microscope. For TCID_50_, 10‐fold serially diluted viruses were mixed with VeroE6/TMPRSS2 cells (2–3 × 10^4^ cells/well) in a 96‐well plate and incubated at 37°C under 5% CO_2_ for 5 days. Five days later, the cytopathic effect in each well was checked, and the TCID_50_ was determined by the Kärber method.^11^

### 2.5 Preparation of hamster antisera and microneutralizing test

The 5‐week‐old female Syrian hamsters were intranasally inoculated with 50 μl of 10^3^ TCID_50_ of each SARS‐CoV‐2 strain: WK‐521 (Pango lineage: A), QH‐329‐037 (B.1), QHN002 (alpha variant, B.1.1.7), TY11‐927 (B.1.617.2), TY11‐908 (delta variant, B.1.617.2), TY38‐873 (BA.1), and TY40‐385 (BA.2). The whole blood was collected by cardiac puncture under deep terminal anesthesia 14–16 days after infection, and sera were prepared by centrifugation. The sera were inactivated at 56°C for 30 min to use for the microneutralization and the PAI test.

Twofold serial dilutions of sera were mixed with 10^2^ TCID_50_ of the SARS‐CoV‐2 strain and preincubated in 96‐well plates at 37°C for 60 min. After preincubation, VeroE6/TMPRSS2 cells (2–3 × 10^4^ cells/well) were added to the virus–serum mixture and incubated at 37°C under 5% CO_2_ for 5 days. Five days later, the cytopathic effect in each well was checked, and the microneutralization titers of sera were determined as the reciprocal of the highest dilution that did not display the cytopathic effect.

### 2.6 PAI test

Prior to performing the PAI test, the nonspecific agglutination factor(s) in antisera was removed if antiserum samples showed nonspecific agglutination as follows. Five hundred microliters of 2.5% hACE2‐beads was centrifuged, and the supernatant was removed. One milliliter of 10‐fold diluted antiserum with PBS was added to the precipitated beads and then mixed by a rotator for 60 min at room temperature. The treated antiserum was centrifuged at 2400 g for 10 min at 4°C, and the collected supernatant was used for the following PAI test.

Twenty‐five‐microliter aliquots of 2‐fold dilution series (from 10‐ to 1280‐fold) of each antiserum were prepared in 96‐well plates with PBS. Four PA units/25 μl of SARS‐CoV‐2 isolates were added to the diluted antisera and incubated for 60 min at room temperature. The accuracy of the PA titer of the added viruses was confirmed by another PA test (back titration). Fifty microliters of 0.01% (w/v) hACE2‐beads were added to each well. After overnight settling at room temperature, the agglutination/agglutination inhibition patterns were observed. For the sedimentation or agglutination control wells, 25 μl PBS was used in place of antisera or virus, respectively.

## 3 RESULTS

### 3.1 Establishment of a PA test

**FIGURE 1:**
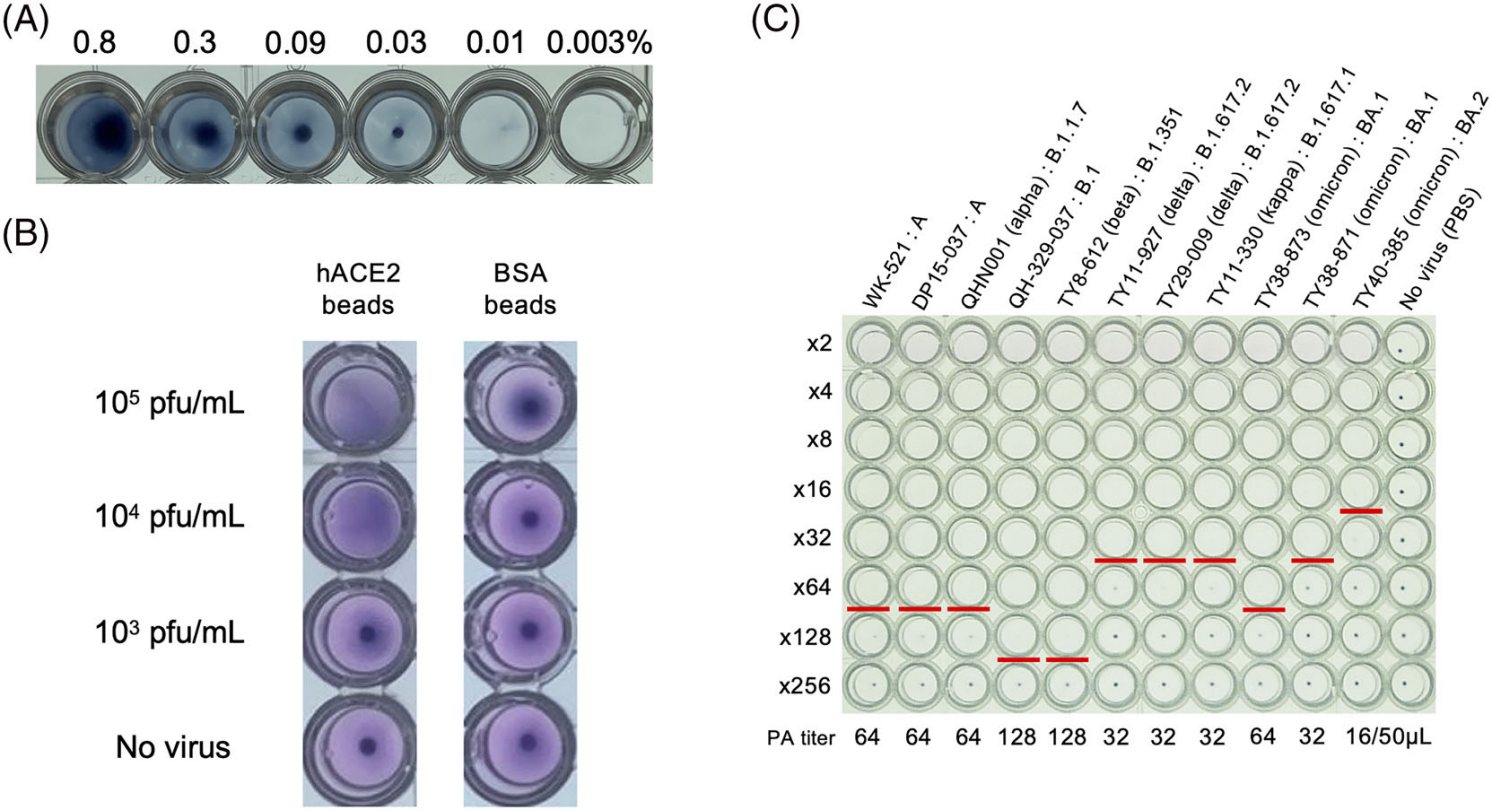
Establishment of the PA test. (A) Optimization of hACE2‐beads concentration. 2.5% hACE2‐beads were diluted from 0.8% to 0.003% by PBS and settled overnight at room temperature. (B) Specificity of the PA test. SARS‐CoV‐2, QH‐329‐037 strain (50 μl) and 0.06% hACE2‐beads/BSA‐beads (50 μl) were mixed and settled overnight at room temperature. (C) The PA test of the SARS‐CoV‐2 variants. A twofold dilution series of the variants (50 μl) was mixed with 0.01% hACE2‐beads (50 μl) and then settled overnight at room temperature. The PA titer was defined as the highest dilution factor at which complete agglutination was observed (red line).

First, we aimed to establish a PA test as a virus titration method using hACE2‐bound latex beads (hACE2‐beads) as a surrogate for the blood cells used in the HA test of influenza viruses. The hACE2‐beads were prepared based on the method used for SARS‐CoV‐2 antigen‐coated latex beads.^12^ A suspension of the prepared beads showed a clear sedimentation pattern after overnight settling at a final concentration of 0.03% (Figure 1A), which was used as the initial condition of the preliminary PA test.

To confirm the specific binding of hACE2‐beads to SARS‐CoV‐2, 50 μl of B.1 isolate was mixed with 50 μl of 0.06% hACE2‐beads (0.03% final concentration) and settled at room temperature. As a control, hACE2‐unbound beads (BSA‐beads) were prepared and tested in the same way as the hACE2‐beads (Figure 1B). A mixture of slight sedimentation and slight agglutination patterns was observed in the virus/hACE2‐beads mixture after 6 h of settling, and the sedimentation and agglutination patterns could be clearly differentiated after overnight settling, whereas only a sedimentation pattern was observed in the virus/BSA‐beads mixture. This suggests that SARS‐CoV‐2 can bind to hACE2‐beads specifically and can be detected by observing the beads. To reduce the settling time required for the assay, another type of manufactured beads with a larger diameter (9.8 μm) was similarly prepared, and the PA test was carried out. However, although the settling time was reduced to <5 h, the 9.8‐μm beads showed only a sedimentation pattern. Therefore, the 0.8‐μm beads were used for the following assays. We also found that the sedimentation patterns were slightly harder to visualize on a V‐bottom microtiter plate compared with a U‐bottom plate, although the settling times on the two plates were almost the same. Therefore, a U‐bottom plate was used for the subsequent assays. Thus, the tentative condition for the PA test was set as follows: mixing 50 μl of virus solution and 50 μl of 0.06% hACE2‐beads (0.03% final concentration), followed by overnight settling at room temperature using a U‐bottom plate. The results did not change when the beads were stored at 4°C for 1 month after preparation.

The PA test was performed on representative SARS‐CoV‐2 isolates under the tentative condition. Agglutination patterns were observed in all tested isolates, and the PA titer of the isolate was defined as the highest dilution factor at which complete agglutination was observed (Figure S1A). However, the BA.2 omicron variant showed the lowest PA titer (2 PA unit/50 μl), which was inadequate to perform the following PAI test. To improve the PA titer, the concentration of hACE2‐beads was re‐examined. The final beads concentration was reduced from 0.03% to 0.005% and 0.0025%, and then the PA titers of three isolates (A, B.1.617.2, and BA.2) were compared among the three beads concentrations (Figure S1B). All PA titers increased with declining hACE2‐beads concentrations, and the final concentration of 0.005% was newly set as the standard condition based on the PA titer and visibility of sedimentation/agglutination patterns. Although sedimentation was not seen even at a final concentration of 0.01% in Figure 1A, this was because the concentration of BSA was different between Figures 1A and S1B. The retest with a 0.005% beads concentration resulted in an eightfold increase in the PA titers of all isolates (Figure 1C). The PA titers of isolates showed a strong positive correlation (r = 0.85) with plaque‐forming units measured by a plaque assay (Figure S2), indicating that the PA titer reflects the amount of virus in the sample. However, the PA test showed different sensitivities among the variants and was the most sensitive to the omicron variants. One of the factors for higher sensitivity in the PA test is probably the affinity of the virus to ACE2. Thus, this phenomenon would be because the omicron variants have a higher affinity for ACE2 than the previous variants.^13^

### 3.2 Establishment of a PAI test

**FIGURE 2:**
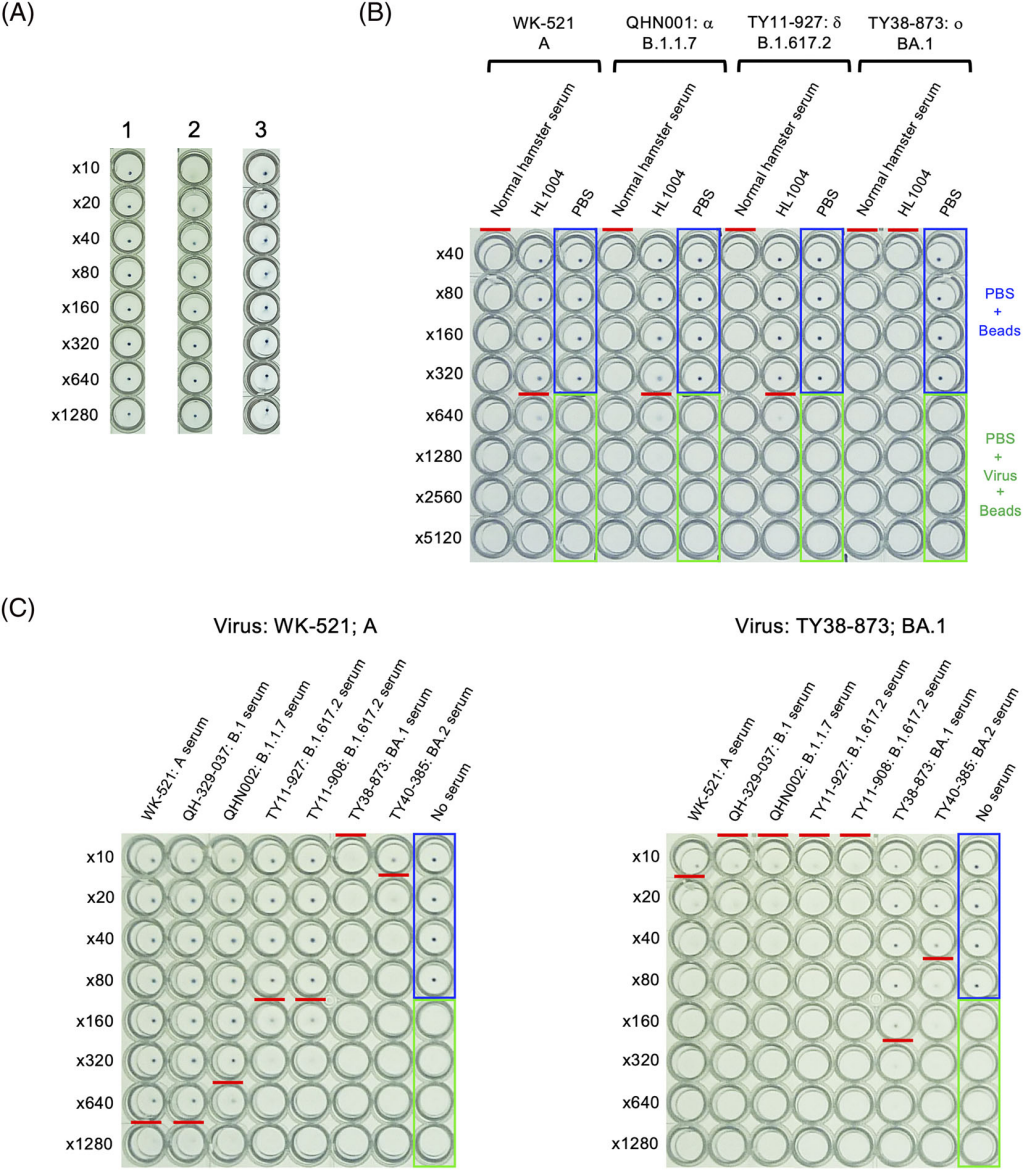
Establishment of the PAI test. (A) The confirmation and removal of nonspecific agglutination factor(s). The serially diluted antisera (25 μl) were mixed with 0.01% hACE2‐beads (25 μl) and then settled overnight at room temperature. 1: Antiserum without the factor(s); 2: antiserum with the factor(s); 3: antiserum after removal of the factor(s). (B) Specificity of the PAI test. The serially diluted serum or antibody (25 μl) was mixed with the isolates (4 PA/25 μl) and allowed to react for 60 min at room temperature. 0.01% hACE2‐beads (50 μl) were added and then settled overnight at room temperature. The wells framed in blue are the sedimentation controls with PBS instead of serum and virus. The wells framed in green are the agglutination controls with PBS instead of serum/antibody. The PAI titer is defined as the highest dilution factor at which complete agglutination inhibition was observed (red line). (C) The PAI test of the SARS‐CoV‐2‐infected antisera. Representative results are shown. The results for WK‐521 (Pango lineage: A) are shown at left, and the results for TY38873 (BA.1) are shown at right.

Next, the inhibitory effect of antibodies against SARS‐CoV‐2 on viral binding to hACE2‐beads was evaluated based on the HI test for influenza virus.^6^ We used antisera obtained from SARS‐CoV‐2‐infected Syrian hamsters because these animals have been shown to be useful as a pathological model of SARS‐CoV‐2 infection, and the antibody titers against SARS‐CoV‐2 were increased in the infected hamsters.^14^ Four hamsters per isolate were inoculated with the isolate (seven isolates in total), and the antisera were prepared. Elevation of antibody titer against the inoculated strain was confirmed by a cell‐based microneutralizing test. The one serum showing the highest antibody titer in the microneutralizing test was selected for the PAI test. As reported in the HI test for influenza virus, nonspecific aggregation factors that interfere with the inhibitory effect of antibodies were observed in some antisera at low dilution factors (Figure 2A, Sample 2). Therefore, prior to the use of such antisera, the factors were removed by pre‐adsorption treatment. The removal of the factor(s) from the antisera was confirmed by mixing the beads (Figure 2A, Sample 3).

The specificity of the binding inhibition was confirmed using a commercially available antibody, HL1004, which does not bind to the omicron variant BA.1 (GeneTex; catalog no. GTX635793: https://www.genetex.com/MarketingMaterial/Index/recombinant_antibodies_for_sars‐cov‐2_research; the web page does not list the reactivity for BA.1, but the same region as for the spike protein B.1.1.529 is used for the reactivity check). A normal (noninfected) Syrian hamster serum (Fujifilm Wako; catalog no. 569‐76331) was also used a non‐inhibitory control (Figure 2B). Four PA units/25 μl of the isolates (A, B.1.1.7, B.1.617.2, and BA.1) was added to 25 μl of a twofold dilution series (from 40‐ to 5120‐fold dilution) of the normal serum or the HL1004 antibody and allowed to react for 60 min at room temperature. Then, 50 μl of 0.01% hACE2‐beads was added (final concentration 0.005%) and settled overnight at room temperature. For the sedimentation or agglutination control, PBS was added in place of the antibody/serum or the isolates. PAI was not observed (all particles were agglutinated) with the normal hamster serum, whereas it was observed with the HL1004 antibody in A, B.1.1.7, and B.1.617.2 isolates, and not in BA.1 isolate as expected. The PAI titer was defined as the highest dilution factor of the serum/antibody for which complete agglutination inhibition was observed. The titers for A, B.1.1.7, B.1.617.2, and BA.1 isolates were 320, 320, 320, and <40, respectively. This suggests that hACE2‐beads can be used for the PAI test under the following standard condition: mixing of 25 μl of serum and 25 μl of virus solution (4 PA unit/25 μl) for 60 min, followed by mixing of 50 μl of 0.01% hACE2‐beads and overnight settling at room temperature.

The PAI test was then performed under the standard condition as defined above (Figure 2C). Agglutination/agglutination inhibition patterns were observed, reflecting the reactivity of the antiserum with the virus. The obtained PAI titers of each antiserum to the tested isolates are summarized in Table 1. The homologous PAI titer was defined as the titer against the same isolate/the same lineage of isolate used to prepare an antiserum as shown in Table 1 (highlighted in italic bold). Anti‐A‐isolate serum showed a homologous PAI titer of 640, whereas the PAI titer was <10 against BA.1 and vice versa: The homologous PAI titer and PAI titer were 160 and 10 against A isolate in anti‐BA.1‐isolate serum. In the HI test for influenza viruses, antigenicity has generally been judged to be different when an HI titer is eightfold or more different from a homologous HI titer. Based on this criterion, we defined an eightfold or greater difference between the PAI titer and homologous PAI titer as an antigenic difference in our PAI test. Our results revealed that A lineage was antigenically different from B.1.351 and BA.1 and BA.2. Similarly, B.1 and B.1.1.7 were antigenically different from BA.1 and BA.2. B.1.617.2 were antigenically different from B.1.351 and BA.1 and BA.2. BA.1 and BA.2 were also antigenically different from the previous variants. This clearly showed that the antigenicity of the omicron (BA.1 and BA.2) and the previous variants/isolates are different.

**TABLE 1:** The PAI titers of SARS‐CoV‐2‐infected hamster antisera

	WK‐521 A serum	QH‐329‐037 B.1 serum	QHN002 Alpha B.1.1.7 serum	TY11‐927 Delta B.1.617.2 serum	TY11‐908 Delta B.1.617.2 serum	TY38‐873 Omicron BA.1 serum	TY40‐385 Omicron BA.2 serum
WK‐521 A	***640***	640	320	80	80	<10	10
DP15‐037 A	***640***	640	320	80	80	<10	10
QH‐329‐037 B.1	640	***640***	640	160	160	<10	10
QHN001 Alpha B.1.1.7	640	640	***640***	80	80	<10	10
TY8‐612 Beta B.1.351	80	320	320	<10	20	< 10	10
TY11‐927 Delta B.1.617.2	320	320	160	***160***	***160***	<10	<10
TY29‐009 Delta B.1.617.2	160	160	160	***80***	***80***	<10	<10
TY11‐330 Kappa B.1.617.1	320	320	160	80	40	<10	<10
TY38‐873 Omicron BA.1	10	<10	<10	<10	<10	***160***	40
TY38‐871 Omicron BA.1	<10	<10	<10	<10	<10	***80***	40
TY40‐385 Omicron BA.2	20	40	40	<10	<10	10	***160***

*Note*: The homologous PAI titers are shown in italic bold.

Antigenic differences between the D614G variant (B.1 lineage) and the omicron variants have been reported in human^15^ or guinea pig^16^ convalescent and vaccinated sera by SARS‐CoV‐2 pseudo‐virus neutralizing assay. Furthermore, the results of the PAI test also suggested antigenic differences between A and B.1.351 and between B.1.351 and B.1.617.2. The antigenic difference of B.1.351 has been reported by a structure–function analysis of the monoclonal antibodies from beta‐variant‐infected individuals.^17^ Collectively, our findings indicate that the PAI test is a useful method for the antigenic analysis of SARS‐CoV‐2 and is easier than the previously available methods.

Comparing the results of the PAI (Table 1) and the microneutralizing (Table 2) tests based on the same criterion, similar results were observed: the antigenic difference between the omicron and the previous variants, the difference between A and B.1.351, and the difference between B.1.617.2 and B.1.351. However, discrepancies were also observed between the tests; the antigenic differences between the following strains were seen in the microneutralizing, not in the PAI test: between A and B.1.1.7, between B.1.617.2 (TY11‐927) and B.1.1.7, and between B.1.617.2 (TY11‐908) and A, B.1, B.1.1.7, B.1.351. In addition, the antigenic difference between B.1.1.7 and BA.2 shown by the PAI test was not observed in the microneutralizing test. This difference is probably due to the different approaches between these tests; the PAI test is evaluated by only the inhibitory effect of antibodies on the virus binding to the receptor, whereas the microneutralizing test is assessed by the inhibitory effect of antibodies on virus growth. Thus, given that the antigenicity affects the receptor binding in many cases, the PAI test revealed the antigenic difference between the omicron and the previous variants with slightly higher accuracy than the microneutralizing test.

**TABLE 2:** The neutralization antibody titers in the microneutralizing test

	WK‐521 A serum	QH‐329‐037 B.1 serum	QHN002 Alpha B.1.1.7 serum	TY11‐927 Delta B.1.617.2 serum	TY11‐908 Delta B.1.617.2 serum	TY38‐873 Omicron BA.1 serum	TY40‐385 Omicron BA.2 serum
WK‐521 A	***160***	640	320	320	80	<10	10
QH‐329‐037 B.1	80	***320***	160	160	80	<10	<10
QHN001 Alpha B.1.1.7	20	320	***320***	80	20	<10	<10
TY8‐612 Beta B.1.351	20	80	320	10	20	<10	10
TY11‐927 Delta B.1.617.2	160	1280	320	***640***	***1280***	<10	10
TY29‐009 Delta B.1.617.2	640	1280	2560	***1280***	***1280***	<10	20
TY38‐873 Omicron BA.1	20	20	40	10	<10	***320***	320
TY40‐385 Omicron BA.2	10	80	80	<10	40	40	***160***

*Note*: The homologous titers are shown in italic bold.

## 4 DISCUSSION

In this study, we established the PA/PAI test using hACE2‐coated beads. The PA titers were correlated with the amount of SARS‐CoV‐2. The PAI titers clearly showed the antigenic differences between the omicron and the previous variants. Furthermore, the titers also suggested antigenic differences of the beta variant from the original Wuhan strain (lineage A) and the delta variant (B.1.617.2). These antigenic differences were supported by the results shown in previous reports.^15^, ^16^, ^17^ The PA/PAI methods allow titration of SARS‐CoV‐2 and antibodies to the virus via a much simpler process than traditional cell‐based assays, such as plaque assay or neutralization assay without special equipment. Although Esmail et al reported an accurate and rapid PA method for SARS‐CoV‐2 antibody testing,^12^ this method requires the additional preparation of recombinant receptor binding domain in the spike protein for each variant to make antigen‐coated beads and a special equipment with software for image analysis. Because our PAI method does not require those, this is more beneficial for the continuous monitoring of antigenicity of field isolates in SARS‐CoV‐2 surveillance.

Previous report^15^ and this study have shown that antigenic properties of the omicron variants are different from the vaccine strain (Wuhan‐Hu‐1 strain). Recently, these two bivalent vaccines have been approved and are in use. Continuous monitoring of the viral antigenicity of field isolates and the holding status of protective antibodies in certain populations will be a crucial component of SARS‐CoV‐2 surveillance. Further review of vaccine strains will also be necessary. The PA/PAI method for SARS‐CoV‐2 established in this study should be a useful tool to obtain informative data for such discussions.

As shown above, the PA/PAI assay is an easier method, but its cost might be a concern. This assay can be performed for ~US$30 per 96‐well plate. This study used commercially available hACE2 to save the time to prepare the protein, which was the most expensive (~US$800/100 μg). If hACE2 can be prepared in‐house, the cost can be further reduced to ~US$1 per 96‐well plate. Finally, we note that a PA/PAI test using inactivated SARS‐CoV‐2 was not performed in this study. If the PA/PAI test were to be validated with inactivated SARS‐CoV‐2, the assay could be used without any restriction due to the biosafety level.

## AUTHOR CONTRIBUTIONS

**Jun Kobayashi:** Conceptualization; data curation; investigation; methodology; writing‐original draft. **Shutoku Matsuyama:** Data curation; funding acquisition; investigation; methodology; writing‐original draft. **Masayuki Shirakura:** Investigation; resources. **Tomoko Arita:** Investigation; resources. **Yasushi Suzuki:** Investigation; resources. **Hideki Asanuma:** Investigation; resources. **Shinji Watanabe:** Conceptualization; data curation; investigation; resources; writing‐review and editing. **Hideki Hasegawa:** Conceptualization; funding acquisition; resources; writing‐review and editing. **Kazuya Nakamura:** Conceptualization; data curation; methodology; supervision; writing‐original draft; writing‐review and editing.

## CONFLICT OF INTEREST

Jun Kobayashi, Shutoku Matsuyama, and Kazuya Nakamura are listed as inventors on Japanese patent application no. 2022‐153084 covering the use of the PA/PAI test submitted by the National Institute of Infectious Diseases.

## ETHICS STATEMENT

All animal experiments were carried out in accordance with the Guide for Animal Experiments Performed at the National Institute of Infectious Diseases (NIID), and were approved by the Animal Care and Use Committee of NIID (Approval No. 122040).

### PEER REVIEW

The peer review history for this article is available at https://publons.com/publon/10.1111/irv.13093.

## Supporting information


**Table S1.** SARS‐CoV‐2 isolates used in this study.
**Figure S1.** Optimization of the PA test.
**a**, The result using 0.03% hACE2‐beads. A 2‐fold dilution series of the viruses (50 μl) was mixed with 0.06% hACE2‐beads (50 μl), and then settled overnight at room temperature. **b**, Optimization of the hACE2‐beads concentration. The final beads concentration was varied from 0.03 ~ 0.0025%. The PA titers are shown with red lines.
**Figure S2.** The correlation between the PA titer and the plaque‐forming units. The regression line is shown by the blue dot line.

## Data Availability

The data that support the findings of this study are openly available in bioRxiv at https://www.biorxiv.org/content/10.1101/2022.09.08.507221v1.
