# Supplementary material for: Use of the particle agglutination/particle agglutination inhibition test for antigenic analysis of SARS‐CoV‐2
Source: Influenza Other Respir Viruses. 2023 Feb 6;17(2):e13093. doi: 10.1111/irv.13093 (PMC9942275; doi:10.1111/irv.13093)

**Supplemental Files**

Table S1. SARS-CoV-2 isolates used in this study.

| Isolate name | Pango lineage | Variants of Concern | Mutations in spike protein |
| --- | --- | --- | --- |
| WK-521 | A |  | None |
| DP15-037 | A |  | None |
| QH-329-037 | B.1 |  | D614G |
| QHN001 | B.1.1.7 | Alpha | H69-V70del, Y144del, N501Y, A570D, D614G, P681H, T716I, S982A, D1118H |
| QHN002 |  |  |  |
| TY8-612 | B.1.351 | Beta | D80A, D215G, L241-A243del, K417N, E484K, N501Y, D614G, A701V |
| TY11-908 | B.1.617.2 | Delta | T19R, E156-F157del, R158G, L452R, T478K, D614G, P681R, D950N |
| TY11-927 |  |  |  |
| TY29-009 |  |  |  |
| TY11-330 | B.1.617.1 | Kappa | T95I, L452R, E484K, D614G, P681R, Q1071H |
| TY38-873 | BA.1 | Omicron | A67V, H69-V70del, T95I, G142-Y144del, Y145D, N211del, L212I, 214EPEins, G339D, S371L, S373P, S375F, K417N, N440K, G446S, S477N, T478K, E484A, Q493R, G496S, Q498R, N501Y, Y505H, T547K, D614G, H655Y, N679K, P681H, N764K, D796Y, N856K, Q954H, N969K, L981F |
| TY38-871 |  |  |  |
| TY40-385 | BA.2 | Omicron | T19I, L24-P26del, A27S, G142D, V213G, G339D, S371F, S373P, S375F, T376A, D405N, R408S, K417N, N440K, S477N, T478K, E484A, Q493R, Q498R, N501Y, Y505H, D614G, H655Y, N679K, P681H, N764K, D796Y, Q954H, N969K |

Figure S1. Optimization of the PA test.


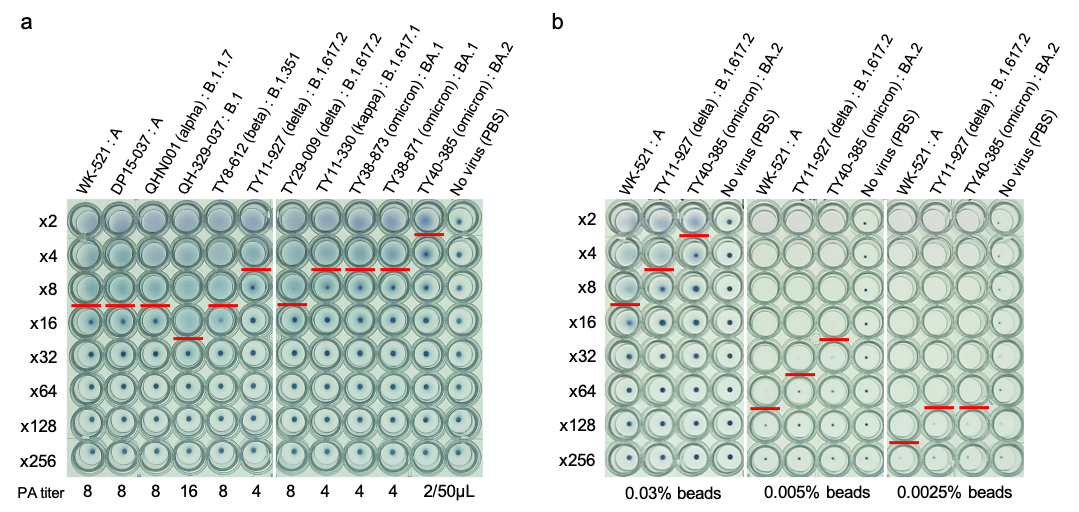


Figure S2. The correlation between the PA titer and the plaque-forming units.


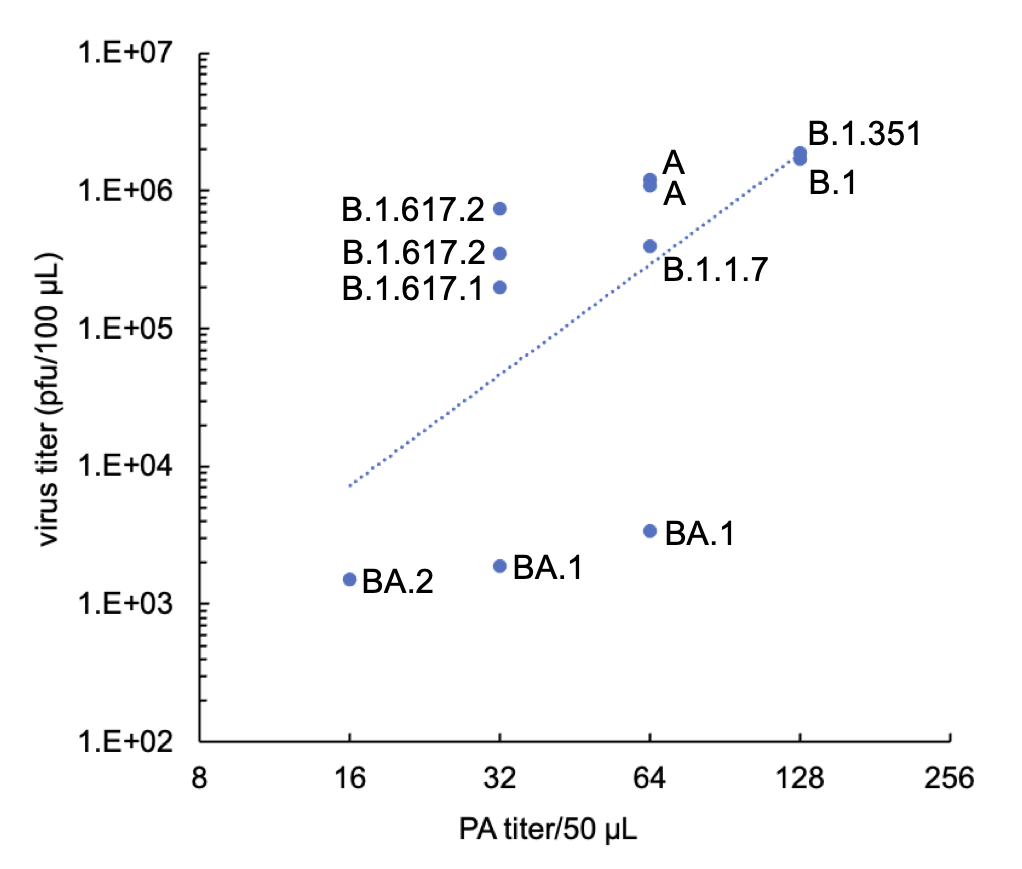

Supplement: Supplementary file 1 — Table S1. SARS‐CoV‐2 isolates used in this study. Figure S1. Optimization of the PA test. a, The result using 0.03% hACE2‐beads. A 2‐fold dilution series of the viruses (50 μl) was mixed with 0.06% hACE2‐beads (50 μl), and then settled overnight at room temperature. b, Optimization of the hACE2‐beads concentration. The final beads concentration was varied from 0.03 ~ 0.0025%. The PA titers are shown with red lines. Figure S2. The correlation between the PA titer and the plaque‐forming units. The regression line is shown by the blue dot line. [file IRV-17-e13093-s001.docx]
